# All-*trans *retinoic acid suppresses interleukin-6 expression in interleukin-1-stimulated synovial fibroblasts by inhibition of ERK_1/2 _pathway independently of RAR activation

**DOI:** 10.1186/ar2569

**Published:** 2008-12-10

**Authors:** Mélanie Kirchmeyer, Meriem Koufany, Sylvie Sebillaud, Patrick Netter, Jean-Yves Jouzeau, Arnaud Bianchi

**Affiliations:** 1 Laboratoire de Physiopathologie et Pharmacologie Articulaires (LPPA), UMR 7561 CNRS-Nancy Université, 54505 Vandœuvre-lès-Nancy, France

## Abstract

**Introduction:**

Interleukin-6 (IL-6) is thought to play a pathogenic role in rheumatoid arthritis and synovium is a major source of IL-6 release. We investigated the ability of retinoids to suppress IL-6 expression in IL-1-stimulated synovial fibroblasts, with special care to the contribution of retinoic acid receptor (RAR) and retinoid X receptor (RXR) subtypes, and the implication of the mitogen-activated protein kinase (MAPK) pathway.

**Methods:**

RAR-α, -β, and -γ and RXR-α, -β, and -γ levels were determined by reverse transcription-quantitative polymerase chain reaction (RT-qPCR) or Western blot in rat synovial fibroblasts stimulated with 10 ng/mL of IL-1β. Stimulated levels of IL-6 were assessed by RT-qPCR or immunoassays in the presence or absence of 1 μM all-*trans *retinoic acid (ATRA) (RAR agonist) or 0.3 μM BMS-649 (RXR agonist). The contribution of RAR subtypes was checked with selective agonists or small interfering RNAs. The effect of ATRA on upstream MAPK (p38 MAPK, c-Jun N-terminal kinase [JNK], and extracellularly regulated kinase 1/2 [ERK_1/2_]) was assessed by Western blot, and the contribution of the ERK_1/2 _pathway to the activation of pro-inflammatory transcription factors was studied by TransAm™ assays.

**Results:**

Synovial fibroblasts expressed all RAR and RXR subtypes except RXR-γ. In IL-1-stimulated cells, ATRA, but not BMS-649, reduced *IL-6 *expression whereas selective RAR agonists were inactive. The inhibitory effect of ATRA on *IL-6 *was not affected by the silencing of RAR subtypes. ATRA also reduced the phosphorylation of ERK_1/2_, but not of p38 MAPK or of JNK. The suppressive effect of ATRA on the activation of activator protein-1 (AP-1) and nuclear factor-IL-6 (NF-IL-6) was reproduced by the MEK1 (mitogen-activated protein extracellularly regulated kinase kinase 1) inhibitor PD-98059, whereas ATRA and PD-98059 had no effect on NF-κB activation.

**Conclusions:**

Among RAR and RXR agonists, only ATRA inhibited IL-1-induced IL-6 expression in rat synovial fibroblasts by inhibiting ERK_1/2 _pathway and subsequent activation of AP-1 and NF-IL-6 independently of RAR.

## Introduction

Retinoids are natural or synthetic analogs of vitamin A, including all-*trans *retinoic acid (ATRA) and its 9-*cis *isomer (9-cis RA). ATRA and other retinoids play a major role in a wide range of physiological pathways such as cell proliferation, embryogenesis, differentiation, morphogenesis, and inflammation (for a review, see [1]). Retinoids exert their functions through their binding to the retinoic acid receptor (RAR) and the retinoid X receptor (RXR), which belong to the subfamily B (respectively, NR1B and NR2B) of the nuclear hormone receptors. Each receptor is divided into three subtypes, which are referred as RAR-α, -β, or -γ and RXR-α, -β, or -γ and which are encoded by separate genes [2]. After binding of retinoids, RAR and RXR form a homodimer or a heterodimer and activate the cellular machinery for an increased transcription rate. But RAR and RXR can alternatively induce gene transrepression by sequestering transcription factors such as activator protein-1 (AP-1) or nuclear factor-interleukin-6 (NF-IL-6) without binding to DNA [2]. Based on the regulatory role of these transcription factors in the control of many inflammatory mediators, liganded RAR complexes can repress a broad spectrum of genes, including inflammatory proteins, cytokines, or matrix metalloproteases (MMPs) [3].

Rheumatoid arthritis (RA) is an immune-mediated inflammatory disease characterized by a chronic inflammation of the synovial membrane which organizes into an aggressive front of tissue able to invade and destroy local articular structures [4]. Although the cause of RA remains unknown, it has been established that cytokine networks play a pivotal role in the immuno-inflammatory and destructive response of RA [5]. Besides tumor necrosis factor-alpha (TNF-α) or IL-1, the pro-inflammatory and pleiotropic cytokine IL-6 could have important activities in the context of pathogenesis of RA [6]. Hence, huge amounts are found in the synovial fluid and tissue and in the sera of arthritic patients [7], and IL-6 serum levels have been correlated with the activity of the disease [6]. IL-6 is synthesized and then secreted extensively by fibroblast-like synoviocytes from RA patients [8,9]. The synthesis is regulated mainly by the transcription factors NF-IL-6, CAAT-enhancer-binding protein (C/EBP)-β, AP-1, and nuclear factor-kappa-B (NF-κB) [8,10,11], which are constitutively activated in RA synovial tissue (for a review, see [12]) and have binding sites in the promoter region of the *IL-6 *gene. Among possible pathogenic roles, IL-6 activates T cells and macrophages, induces osteoclast differentiation, causes systemic inflammatory manifestations, and could promote angiogenesis [6]. As a consequence, the blockade of IL-6 effects has emerged as a new therapeutic approach to RA, and tocilizumab, a humanized anti-human IL-6 receptor monoclonal antibody, has successfully entered the clinics (for a review, see [13]). These clinical data have confirmed the pathological role of IL-6 in RA (for a review, see [13]) and suggest that this second generation of anti-cytokine therapy may have therapeutical relevance in patients who have a limited response to disease modifying anti-rheumatic drugs or biological agents, such as inhibitors of TNF-α [5].

Beside their successful use in the treatment of skin diseases or cancer, retinoids were shown to be anti-inflammatory in several animal models of RA. Thus, a decrease of cartilage lesions, associated with a reduction of *MMP-1 *expression, was reported in the paws of adjuvant arthritis (AA) rats treated with 13-cis RA [14]. In the rodent collagen-induced arthritis (CIA) model, ATRA improves the course of the disease and reduces the production of inflammatory cytokines [15], and Am-80 (RAR agonist) decreases anti-collagen II antibody levels and improves joint swelling and bone destruction [16]. However, in contrast to its efficacy in the AA model, 13-cis RA remained poorly effective in the rat CIA model [16]. Apart from differences in the pathogenic mechanisms of animal models of RA [17] or in the binding activity of retinoids to RAR subtypes, these experimental data strongly suggest that the anti-arthritic effect of RAR agonists is supported mainly by their ability to reduce the immune response. The inflamed synovial tissue is a major source of IL-6 production [18], and the blockade of IL-6 [19] or its deficiency [20] reduced the severity of experimental arthritis by impairing the T-cell response. These data led us to postulate that the ability of retinoids to inhibit IL-6 production by activated synovial fibroblasts could have a major role in their antiarthritic potency. Therefore, we compared the suppressive properties of the RAR agonist ATRA and of the RXR agonist, BMS-649, on IL-1-induced expression of IL-6 in rat synovial fibroblasts and tried to elucidate the corresponding molecular events with respect to the contribution of RAR/RXR subtypes and signalling pathways.

First, we used compounds acting either on RAR or RXR, alone or in combination, to demonstrate that the downregulation of IL-6 was not dependent of RXR activation. Using mRNA silencing of RAR subtypes and selective agonists of RAR-α, -β, or -γ, we showed that the inhibitory potency of ATRA on IL-6 was independent of RAR activation. Finally, we demonstrated that the MEK1 (mitogen-activated protein extracellularly regulated kinase kinase 1) inhibitor PD-98059 reproduced the ability of ATRA to reduce the IL-1-induced phosphorylation of extracellularly regulated kinase 1/2 (ERK_1/2_) and activation of AP-1 and of NF-IL-6, but not of NF-κB. These data demonstrate that ATRA suppressed the IL-1-induced production of IL-6 in an RAR-independent manner by inhibiting the ERK_1/2 _pathway and subsequent activation of AP-1 and NF-IL-6 in synovial fibroblasts.

## Materials and methods

### Synovial fibroblast isolation and culture

Rat synovial fibroblasts were obtained from synovium collected aseptically from healthy Wistar male rats (130 to 150 g; Charles River, L'Arbresle, France), killed under dissociative anesthesia (ketamine [Rhône-Mérieux, Lyon, France] and acepromazine [Sanofi Santé Animale, Paris, France]) in accordance with national animal care guidelines, after approval by our internal ethics committee, as described before [21]. Cells were cultured as monolayers in 75-cm^2 ^flasks at 37°C in a humidified atmosphere containing 5% of CO_2_, until the fourth passage, in order to prevent any strong contamination by macrophage-like cells and to obtain a homogenous population of synovial fibroblasts. The cell phenotype was controlled by measuring the mRNA level of the fibroblast-like markers synoviolin and cadherin-11 [22] and of the macrophage-like marker CD14 [23].

### Study design

The expression of IL-6 was studied in rat synovial fibroblasts cultured under low fetal calf serum conditions and stimulated with 10 ng/mL of rat recombinant IL-1β (R&D Systems, Lille, France) in the presence or absence of a RAR agonist, ATRA (Sigma-Aldrich, St. Louis, MO, USA), used at 1 μM and/or an RXR agonist, BMS-649 (kindly provided by Bristol-Myers Squibb Company, Princeton, NJ, USA), used at 0.3 μM. Selective agonists (kindly provided by Bristol-Myers Squibb Company) of RAR-α (BMS-753), RAR-β (BMS-453), or RAR-γ (BMS-961) were used at 0.1 or 1 μM. All drugs were maintained in the dark before addition to the culture medium, at a final concentration of 0.1% or 0.2% of dimethyl sulfoxide (DMSO), at the same time as IL-1 stimulation. The contribution of RAR subtypes to the effect of ATRA was investigated using the RNA interference technology. The effect of ATRA on the activation of mitogen-activated protein kinase (MAPK) pathways was studied in IL-1-stimulated cells by measuring the phosphorylation of p38 MAPK, stress-activated protein kinase/c-Jun N-terminal kinase (SAPK/JNK), or ERK_1/2 _by Western blot after 5, 15, or 30 minutes, respectively. The effect of ATRA and the possible contribution of ERK_1/2 _on IL-1β-induced activation of NF-κB, NF-IL-6, or AP-1 were studied by TransAm™ assays (Active Motif, Rixensart, Belgium).

### Assay for synovial fibroblast viability

Cell viability was assessed by the mitochondria-dependent reduction of 3-(4,5-dimethylthiazol-2-yl)-2,5-diphenyl-2*H*-tetrazolium bromide (MTT) (Sigma-Aldrich) as described before [24].

### Gene-silencing experiment

For experiments of RAR subtype silencing, double-stranded small interfering RNA (siRNA) corresponding to a region of RAR-α (GCAGUUCGGAAGAGAUAGU), RAR-β (CUAGCUACUGGCUUCAAGA), or RAR-γ (GCCUUCUUCCUUUACCUCC) mRNA was synthesized by Eurogentec (Liege, Belgium). Transfection of synovial fibroblasts with 50 nM siRNA was performed during 24 hours using X-TremeGENE reagent^® ^(Roche Molecular Biochemicals, Meylan, France) in accordance with manufacturer recommendations. Cells were then stimulated with IL-1β for 6 hours in the presence or absence (DMSO alone) of ATRA.

### RNA extraction and reverse transcription-polymerase chain reaction analysis

After stimulation with IL-1β in the presence or absence (DMSO alone) of RAR or RXR agonists, total RNA was extracted from cell layers using an RNeasy extraction kit in accordance with the recommendations of the manufacturer (Qiagen, Courtabœuf, France), as described before [21]. The mRNA levels for *RAR-α*, -*β*, or -*γ*, *RXR-α*, -*β*, or -*γ*, *IL-6*, and ribosomal protein S29 (*RP29*) were quantified by real-time quantitative polymerase chain reaction (PCR) in capillaries with the Lightcycler™ technology (Roche Molecular Biochemicals). The reaction mixture was composed of cDNAs, SYBR green master mix system^® ^(Qiagen), and primer pairs specific for the DNA fragments to be amplified. The sequences of the primers used were *IL-6 *(sense: 5'-CCGGAGAGGAGACTTCACAG-3', anti-sense: 5'-CCGGAGAGGAGACTTCACAG-3', NM 012589, 161 base pairs [bp], 59°C), *RAR-α *(sense: 5'-ACCAGATTACCCTTCTCAAGG-3', anti-sense: 5'-GGTACCGCGTGCAGATT-3', NM 031528, 65 bp, 58°C), *RAR-β *(5'-TCTTAATGAAAATCACAGATCTCCGC-3', anti-sense: 5'-CCAGGAATTTCCATTTTCAAGG-3', XM 223843, 62 bp, 57°C), *RAR-γ *(sense: 5'-AGTGCTATCTGCCTCATCT-3', anti-sense: 5'-TTGTCCACCTTCACCTTCTCGGGTTC-3', NM 001062412, 66 bp, 62°C), *RXR-α *(5': GAAGCGTACTGCAAACACAAG-3', anti-sense: 5'-CAGCCGGAGCAGCAGCTTGG-3', NM 012805, 65 bp, 66°C), *RXR-β *(sense: 5'-CTTCATGTGCACAGAAACT-3', anti-sense: 5'-TCTGTCAGCACCCGATCAAA-3', NM 206849, 68 bp, 55°C), *RXR-γ *(sense: 5'-CTGCACCGGGCAGGGTGGAAT-3', anti-sense: 5'-CTGGACGGAAACCGAGCGGTG-3', NM 031765, 65 bp, 66°C), and *RP29 *(sense: AAGATGGGTCACCAGCAGCTCTACTG-3', anti-sense: 5'-AGACGCGGCAAGAGCGAGAA-3', NM 012876, 70 bp, 59°C).

The quantity of each cDNA was estimated by threshold cycle (Ct), defined as the number of cycles corresponding to the moment when the fluorescence, secondary to SYBR green incorporation into double-stranded DNA, became detectable at the end of the PCR elongation phase. At completion of PCR cycling, a melting curve was performed in order to control the specificity of each amplified product. Each experiment included positive and negative reaction controls, and Ct values were converted into nanograms of DNA using calibration curves made of serial dilutions of known amounts of corresponding purified PCR products. The transcript level of the housekeeping gene *RP29 *was determined in parallel for each sample, and data were expressed as the normalized ratio of mRNA level of each gene of interest over the *RP29 *gene.

### Measurement of IL-6 production

IL-6 secreted in culture supernatant after 24 hours of incubation was measured in nanograms per milliliter with commercially available rat enzyme-linked immunosorbent assay (ELISA) (R&D Systems, Abingdon, UK) with a limit of detection of 21 pg/mL. The assay was not sensitive to soluble receptors and no cross-reactivity was reported with other cytokines (manufacturer's data). Positive controls were used in each experiment.

### Western blot analysis

For protein study, experiments were performed from 5 minutes to 24 hours after IL-1β challenge. Cellular proteins were extracted with 1× Laemmli blue (Sigma-Aldrich), as described before [24], and electroblotted with RAR-α, RAR-β, RAR-γ, RXR-α, RXR-β, or RXR-γ (1:2,000; Santa Cruz Biotechnology, Le Perray en Yvelines, France), phospho-ERK_1/2 _(P-ERK_1/2_), phospho-p38 MAPK (P-p38 MAPK) (1:1,000; Cell Signaling Technology, Inc., Danvers, MA, USA), phospho-SAPK/JNK (P-JNK) (1:500; Cell Signaling Technology, Inc.), or β-actin (1:4,000; Sigma-Aldrich). Detection of protein bands was done by chemiluminescence with the Phototope Detection system in accordance with the recommendations of the manufacturer (Cell Signaling Technology, Inc.). Results were expressed as the ratio of each protein of interest over β-actin, used as internal control.

### ELISA-based TransAm™ kit for NF-IL-6, NF-κB (p65), and AP-1 (c-Jun)

Synovial fibroblasts were preincubated for 1 hour with 10 μM PD-98059 (inhibitor of ERK_1/2_; Sigma-Aldrich) or incubated with 1 μM ATRA before stimulation for 30 minutes or 4 hours with IL-1β. At corresponding times, nuclear extracts were prepared with a commercially available kit according to the recommendations of the manufacturer (Active Motif), as described before [24]. After protein quantification with a bicinchoninic acid assay kit (BCA™ protein assay kit; Sigma-Aldrich), nuclear extracts were checked for NF-IL-6, NF-κB (p65), and AP-1 (c-Jun) activation using ELISA-based kits (TransAm™). Briefly, 2 μg of nuclear proteins was added in a volume of 20 μL to a well plate precoated with oligonucleotides (5'-GGGACTTTCC-3') corresponding to NF-κB responsive element (RE), or (5'-GCAAT-3') corresponding to NF-IL-6 RE, or (5'-TGAGTCA-3') corresponding to AP-1 RE. Wells were then incubated with a primary antibody against phosphorylated NF-κB (p65) (1:1,000), phosphorylated NF-IL-6 (1:1,500), or phosphorylated AP-1 (c-Jun) (1:500). The RE/NF-κB, NF-IL-6, or AP-1 antibody complex was revealed by incubation with a secondary horseradish peroxidase-conjugated antibody. The amounts of NF-κB (p65), NF-IL-6, or AP-1 (c-Jun) proteins in nuclear extracts were quantified by measuring the absorbance at 450 nm on a Multiskan™ microplate reader (Labsystems, part of Thermo Fisher Scientific Inc., Waltham, MA, USA). The specificity of the assay was controlled by demonstrating that the addition of an excess of wild-type consensus RE (20 pmol/well) prevented transcription factor binding onto the well whereas an excess of mutated consensus RE remained ineffective.

### Statistical analysis

Data are expressed as the mean ± standard deviation of at least three independent assays. Comparisons were made by analysis of variance, followed by Fisher protected least-squares difference *post hoc *test, using Statview™ version 5.0 software (SAS Institute Inc., Cary, NC, USA). A *P *value of less than 0.05 was considered significant (*P < 0.05 versus control and ^#^*P *< 0.05 versus IL-1β).

## Results

### 1. Effect of IL-1β on the expression of RAR and RXR subtypes

**Figure 1:**
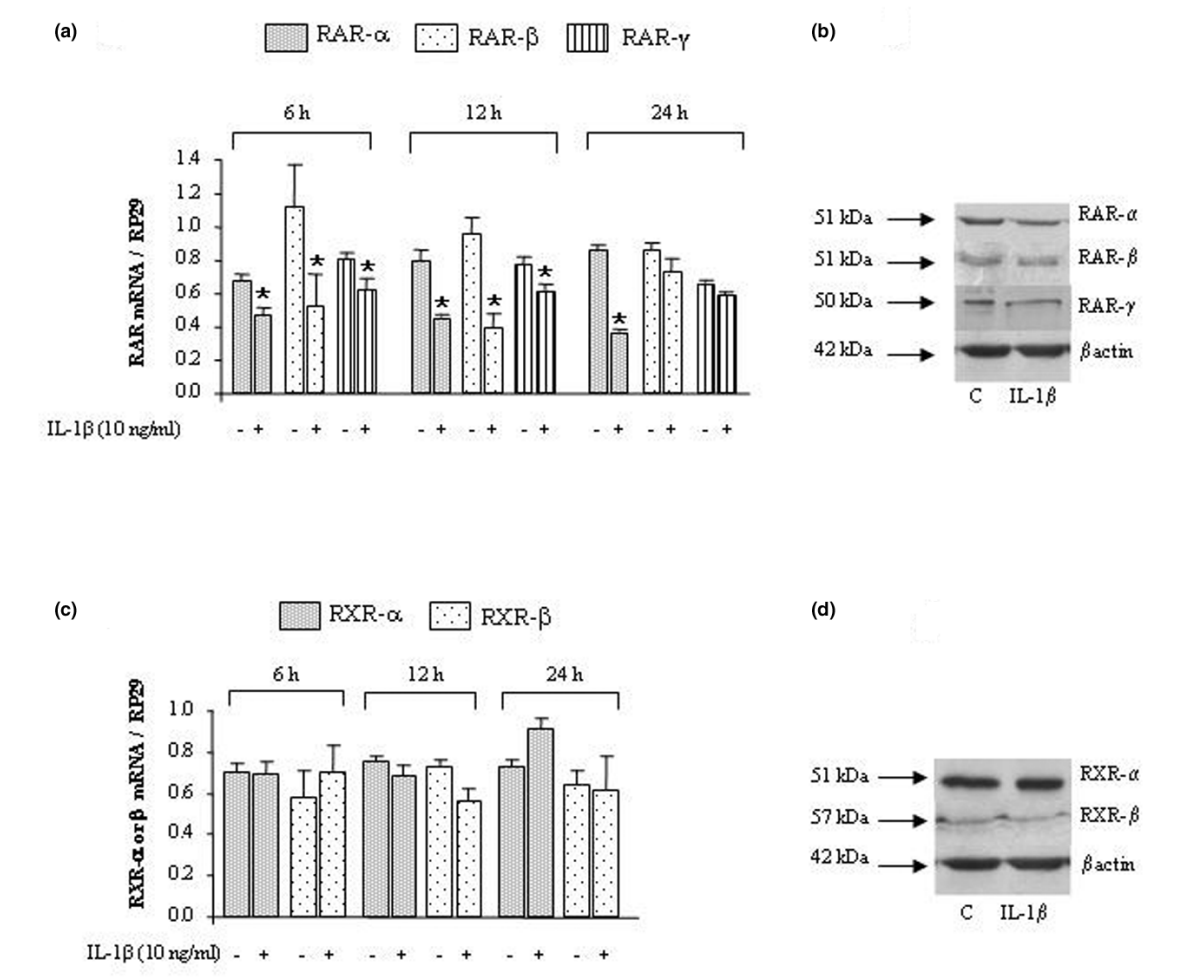
**Effect of interleukin-1-beta (IL-1β) on the expression of retinoic acid receptor (RAR) or retinoid X receptor (RXR) subtypes in synovial fibroblasts**. Rat cells were stimulated with 10 ng/mL of IL-1β for 6, 12, or 24 hours for *RAR *or *RXR *gene expression and for 24 hours (C for control) for RAR or RXR protein expression. Levels of mRNA of *RAR *(α, β, γ) **(a) **or *RXR *(α, β, γ) **(c) **quantified by real-time polymerase chain reaction and normalized to ribosomal protein S29 (*RP29*) are shown. Data are expressed as mean ± standard deviation from three independent experiments. Protein expressions of RAR (α, β, γ) **(b) **or RXR (α, β) **(d) **normalized to β-actin are shown. Statistically significant differences from the control are indicated as *P < 0.05.

Preliminary experiments using MTT assay showed no loss of cell viability in rat synovial fibroblasts stimulated with IL-1β (10 ng/mL) in the presence or absence of RAR or RXR agonists at the concentrations used (data not shown). As shown in Figure 1, the mRNAs and the proteins for all RAR subtypes (Figure 1a,b) and for RXR-α and -β (Figure 1c,d) were detected in synovial fibroblasts under basal conditions. The mRNA of *RXR-γ *was not detected in our cell population, but a control experiment showed that it was highly expressed in rat adipose tissue, cortex, and heart (data not shown), therefore confirming its restricted expression profile. In response to a stimulation of 6 to 24 hours with IL-1β, the mRNA levels of *RAR *decreased from 30% to 55% for the -*α *subtype, from 15% to 60% for the -*β *subtype, and from 10% to 25% for the -*γ *subtype (Figure 1a). Levels of mRNA of *RXR-α *or -*β *were not affected by IL-1β stimulation (Figure 1c). Western blotting confirmed these expression profiles at the protein level (Figure 1b,d).

### 2. Effect of retinoids on IL-1β-induced expression of IL-6

**Figure 2:**
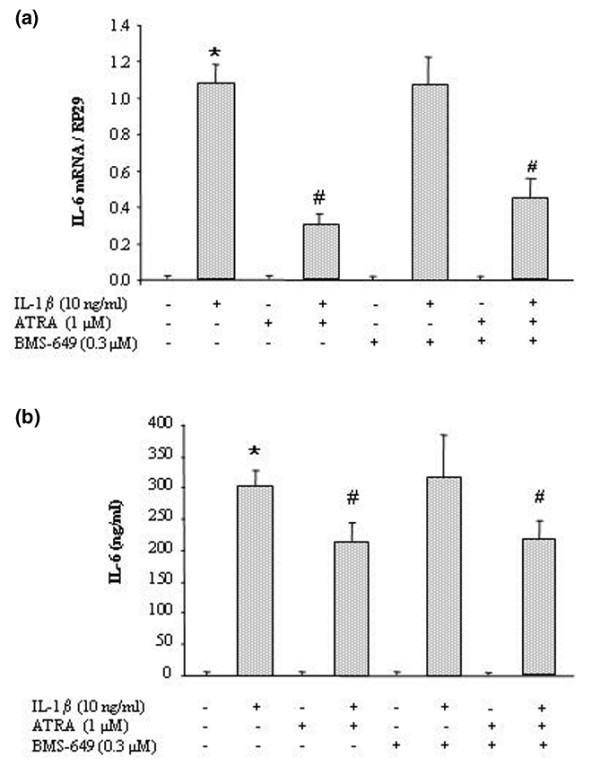
**Effect of retinoids on interleukin-1-beta (IL-1β)-induced expression of IL-6 in synovial fibroblasts**. Rat cells were stimulated with 10 ng/mL of IL-1β for 6 hours (*IL-6 *expression) or 24 hours (IL-6 production) in the presence or absence of 1 μM all-*trans *retinoic acid (ATRA) (retinoic acid receptor agonist) or 0.3 μM BMS-649 (retinoid X receptor agonist). **(a) ***IL-6 *mRNA levels quantified by real-time polymerase chain reaction and normalized to ribosomal protein S29 (*RP29*). **(b) **IL-6 levels (nanograms per milliliter) in culture supernatant by enzyme-linked immunosorbent assay. Data are expressed as mean ± standard deviation of values from at least three independent experiments. Statistically significant differences from the control are indicated as *P < 0.05 and from IL-1β-stimulated cells as ^#^*P *< 0.05.

As shown in Figure 2, *IL-6 *mRNA level (Figure 2a) and corresponding mediator (Figure 2b) were not detected in basal conditions but were strongly expressed in response to IL-1β. In IL-1-stimulated synovial fibroblasts, ATRA (RAR agonist) decreased *IL-6 *gene expression from 70% (Figure 2a) and IL-6 level from 30% (Figure 2b) compared with IL-1β controls. This inhibition was dose-dependent for ATRA between 1 nM and 10 μM (data not shown). In contrast, BMS-649 (RXR agonist) was ineffective on IL-6 expression or production (Figure 2a,b). A costimulation with ATRA and BMS-649 inhibited proinflammatory cytokine expression to the same extent as ATRA alone.

### 3. Effect of RAR-selective agonists on IL-1-induced expression of IL-6

**Figure 3:**
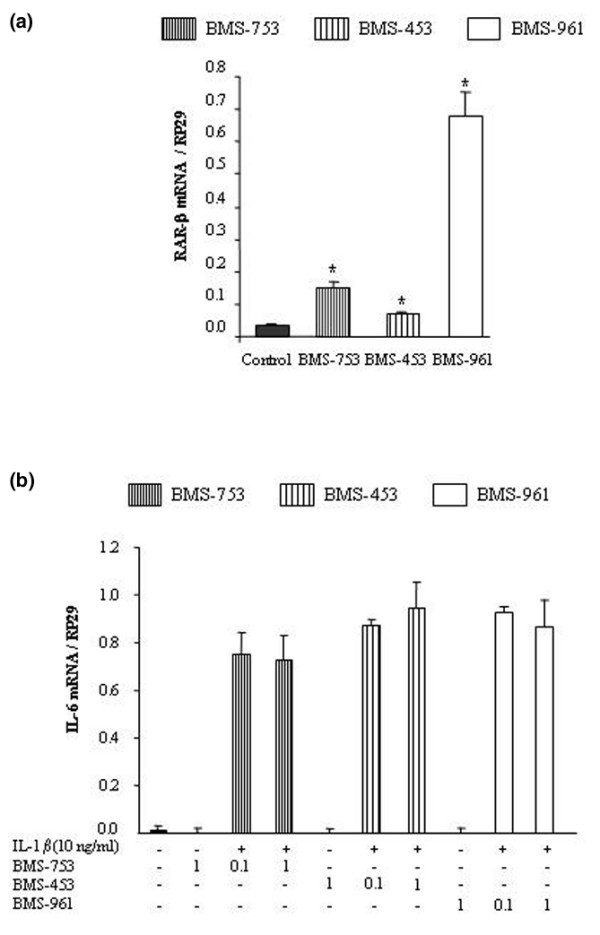
**Effect of selective retinoic acid receptor (RAR) agonists on interleukin-1 (IL-1)-induced expression of IL-6**. **(a) **To control the ability of the selective agonists to trigger RAR-dependent responses, rat synovial fibroblasts were stimulated for 6 hours with 0.1 μM of RAR agonist (BMS-753 for RAR-α, BMS-453 for RAR-β, or BMS-961 for RAR-γ), and the mRNA level of the target gene *RAR-β *normalized to ribosomal protein S29 (*RP29*) was studied by real-time polymerase chain reaction (PCR). **(b) **The suppressive effect of RAR-selective agonists on the *IL-6 *mRNA level normalized to *RP29 *was studied by real-time PCR in cells stimulated with 10 ng/mL of IL-1β in the presence or absence of 0.1 or 1 μM of RAR agonist. Data are expressed as mean ± standard deviation of values from at least three independent experiments. Statistically significant differences from the control are indicated as *P < 0.05.

As the RXR agonist (BMS-649) was inactive on IL-1-induced expression of IL-6 whereas the RAR agonist (ATRA) was inhibitory, we next investigated the contribution of the different RAR subtypes. At the concentration used, the three selective RAR agonists (BMS-753 for RAR-α, BMS-453 for RAR-β, and BMS-961 for RAR-γ) increased the mRNA level of *RAR-β*, chosen as an RAR-dependent target gene, from 2- to 20-fold (Figure 3a). This demonstrated that the RAR-selective agonists were active in our experimental system. A subsequent dose-ranging study demonstrated that none of the three agonists reproduced the inhibitory effect of 1 μM ATRA on IL-6 (Figure 3b). These data demonstrated that RAR subtype activation was not implied in the inhibitory effects of ATRA.

### 4. Effect of RAR silencing on the IL-6-suppressive effect of ATRA

**Figure 4:**
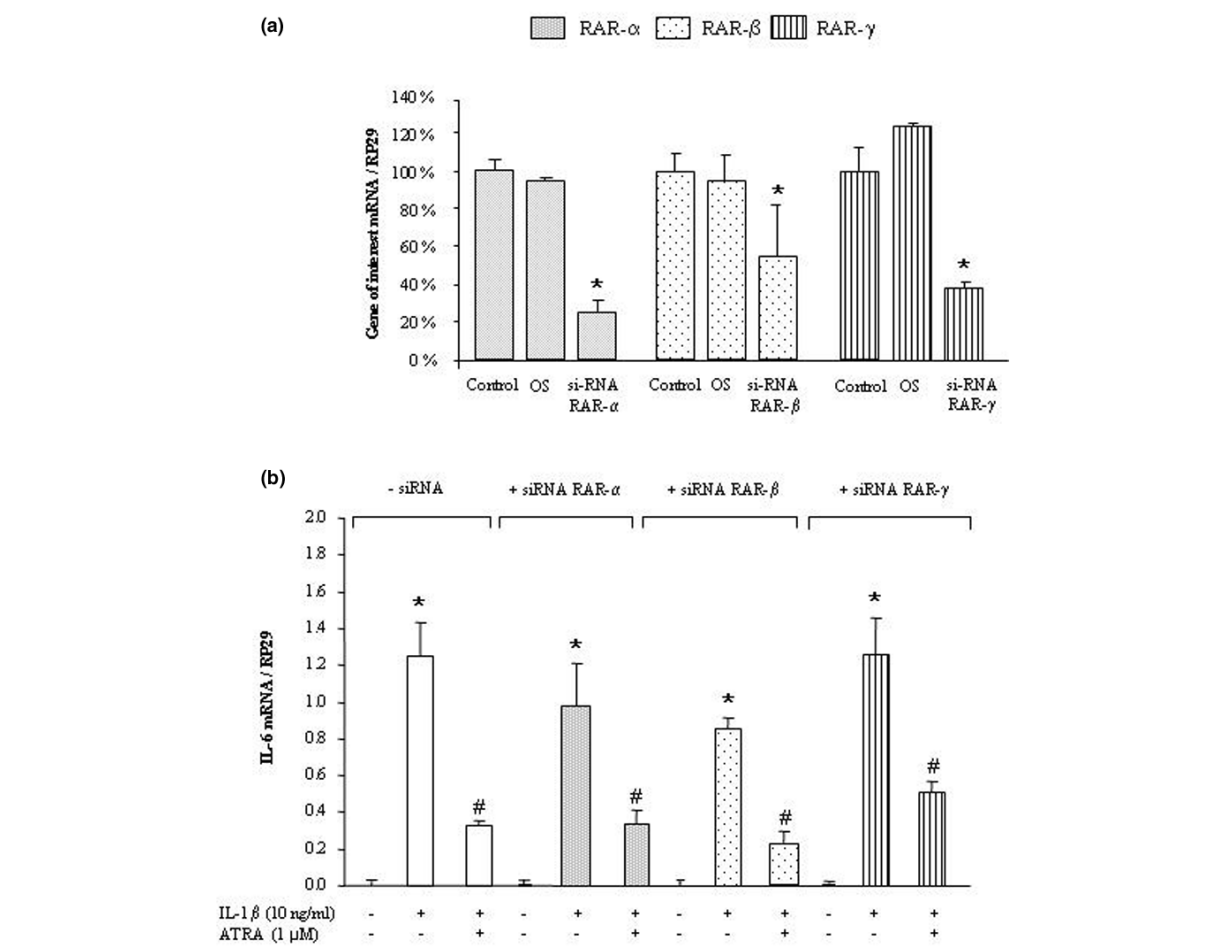
**Effect of retinoic acid receptor (RAR) silencing on the suppressive effect of all-*trans *retinoic acid (ATRA) on interleukin-6 (IL-6) expression**. **(a) **Normalized mRNA levels of *RAR-α, -β*, or -*γ *in rat synovial fibroblasts transfected for 24 hours with 50 nM of small interfering RNA (siRNA) against the corresponding RAR subtype or with 50 nM of an oligonucleotidic sequence (OS), before stimulation with 1 μM ATRA for 6 hours. **(b) **Normalized mRNA levels of *IL-6 *in synovial fibroblasts transfected for 24 hours with 50 nM of each RAR siRNA and then stimulated for 6 hours with 10 ng/mL of IL-1β in the presence or absence of 1 μM ATRA. Data are expressed as mean ± standard deviation of values from at least three independent experiments. Statistically significant differences from the control are indicated as *P < 0.05 and from IL-1β-stimulated cells as ^#^*P *< 0.05. *RP29*, ribosomal protein S29.

siRNAs designed against RAR-α, RAR-β, or RAR-γ were used to confirm whether the cytokine-suppressive effect of ATRA on IL-6 was RAR-independent. As shown in Figure 4a, the transfection of synovial fibroblasts with each siRNA significantly reduced the mRNA level of the targeted RAR subtype, whereas the transfection of an oligomeric sequence has no effect (Figure 4a). After 24 hours of incubation, transfected synovial fibroblasts remained responsive to IL-1β, although the extent of IL-6 induction was slightly lower than in non-transfected cells (Figure 4b). However, whatever the RAR siRNA used, ATRA still inhibited IL-1-induced expression of IL-6 with an efficacy similar to that of non-transfected cells (around 70%) (Figure 4b). These data indicated that the suppressive effect of ATRA on IL-1-induced responses was likely RAR-independent.

### 5. Effect of ATRA on IL-1β-induced activation of MAPK pathways

**Figure 5:**
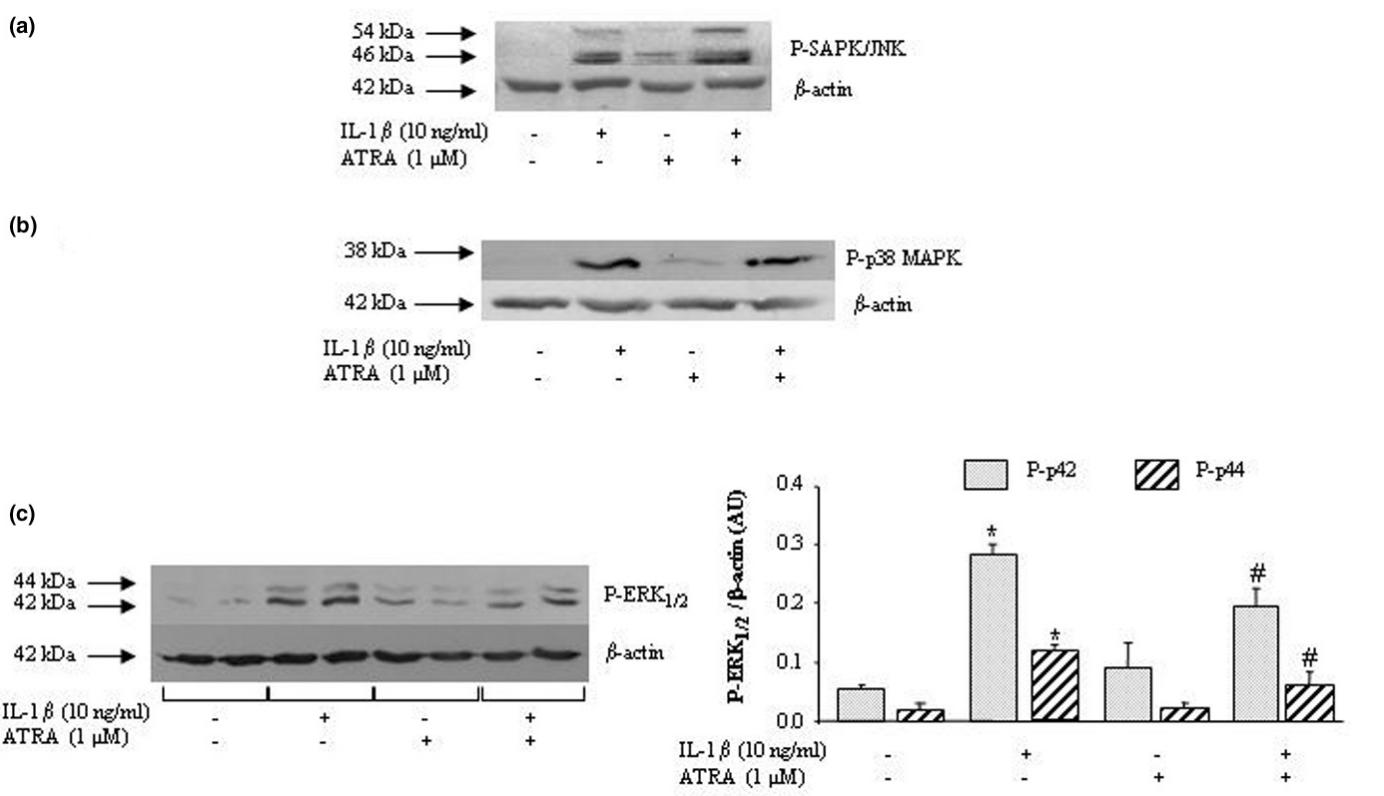
**Effect of all-*trans *retinoic acid (ATRA) on interleukin-1-beta (IL-1β)-induced activation of MAPK pathways in rat synovial fibroblasts**. **(a) **Phospho-JNK (P-JNK) levels after 15 minutes of stimulation with 10 ng/mL of IL-1β in the presence or absence of 1 μM ATRA. **(b) **Phospho-p38 MAPK (P-p38 MAPK) levels after 5 minutes of stimulation with 10 ng/mL of IL-1β in the presence or absence of 1 μM ATRA. **(c) **Phospho-ERK_1/2 _(P-ERK_1/2_, or P-p42/44) levels after 30 minutes of stimulation with 10 ng/mL of IL-1β in the presence or absence of 1 μM ATRA. Data are expressed in arbitrary units (AU) as mean ± standard deviation of protein levels normalized to β-actin collected from at least three independent experiments. Statistically significant differences from the control are indicated as *P < 0.05 and from IL-1β-stimulated cells as ^#^*P *< 0.05. ERK_1/2_, extracellularly regulated kinase 1/2; JNK, c-Jun N-terminal kinase; MAPK, mitogen-activated protein kinase; SAPK, stress-activated protein kinase.

In our experimental system, p38 MAPK, JNK, and ERK_1/2 _pathways were activated by IL-1β, with the phosphorylation of JNK and p38 MAPK being obvious from 5 to 15 minutes and of ERK_1/2 _from 5 to 30 minutes after IL-1β challenge (data not shown). In these experimental conditions, ATRA affected the early phosphorylation neither of p38 MAPK (Figure 5a) nor of JNK (Figure 5b) induced by IL-1β. In contrast, ATRA reduced the level of ERK_1/2 _phospho-proteins from 30% to 50% (Figure 5c). These data demonstrated that, among IL-1-activated MAPK pathways, ATRA was inhibitory only on ERK_1/2 _in rat synovial fibroblasts.

### 6. Contribution of ERK_1/2 _to the activation of AP-1 and NF-IL-6 but not of NF-κB

**Figure 6:**
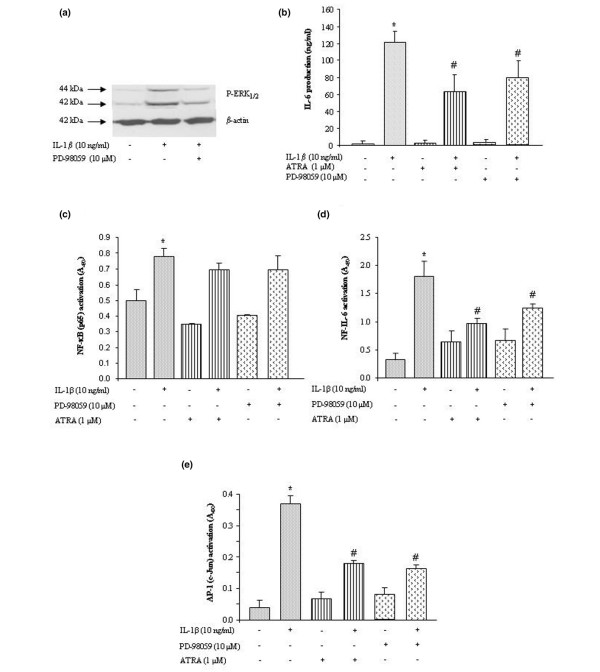
**Contribution of ERK_1/2 _pathway to the inhibitory effect of all-*trans *retinoic acid (ATRA)**. Rat synovial fibroblasts were stimulated with 10 ng/mL of interleukin-1-beta (IL-1β) in the presence or absence of the ERK_1/2 _inhibitor PD-98059 or ATRA. **(a) **Western blotting of phosphorylated ERK_1/2 _(P-ERK_1/2_) after 15 minutes of stimulation in the presence or absence of 10 μM PD-98059. **(b) **Production of IL-6 (nanograms per milliliter by enzyme-linked immunosorbent assay [ELISA]) in culture supernatant after 24 hours of stimulation in the presence or absence of 10 μM PD-98059 or 1 μM ATRA. **(c) **Nuclear content of nuclear factor-kappa-B (NF-κB) by ELISA-based TransAm™ kit after 30 minutes of stimulation in the presence or absence of 10 μM PD-98059 or 1 μM ATRA. Nuclear content of nuclear factor of interleukin-6 (NF-IL-6) **(d) **or activator protein-1 (AP-1) (c-Jun) **(e) **by ELISA-based TransAm™ kit after 4 hours of stimulation in the presence or absence of 10 μM PD-98059 or 1 μM ATRA. Data are expressed as mean ± standard deviation of IL-6 levels or absorbance value at 450 nm values from at least three independent experiments. Statistically significant differences from the control are indicated as *P < 0.05 and from IL-1β-stimulated cells as ^#^*P *< 0.05. ERK_1/2_, extracellularly regulated kinase 1/2.

A control experiment showed that, at the concentration used, PD-98059 efficiently prevented the IL-1β-induced phosphorylation of ERK_1/2 _in our experimental system (Figure 6a). In these conditions, the stimulating effect of IL-1β on IL-6 release was reduced to the same extent (40% to 50%) by PD-98059 and ATRA (Figure 6b). In preliminary experiments, activation of transcriptions factors by IL-1β was shown to be significant after 30 minutes of stimulation for NF-κB (1.6-fold) and after 4 hours of stimulation for NF-IL-6 (5-fold) or AP-1 (c-Jun) (7-fold) (data not shown). In IL-1β-stimulated cells, neither PD-98059 nor ATRA reduced the activation of the NF-κB pathway (Figure 6c), whereas a comparable inhibition (40% to 55%) of NF-IL-6 (Figure 6d) or AP-1 (c-Jun) (Figure 6e) was observed with both molecules. These data suggested that inhibition of the ERK_1/2 _pathway by ATRA contributed to its inhibitory effect on IL-1-induced activation of NF-IL-6 and AP-1 and subsequent production of IL-6.

## Discussion

In the present work, we demonstrated for the first time that RAR and RXR subtypes were expressed in rat synovial fibroblasts at either the mRNA or the protein level. All receptor subtypes, except RXR-γ, were detected and this was consistent with the recent demonstration that their mRNAs, except the *RXR-γ *mRNA, were expressed in human osteoarthritic chondrocytes [25]. In response to IL-1 stimulation, the expression of all RAR subtypes decreased in synovial fibroblasts, whereas those of RXR-α and RXR-β remained unaffected. However, such inhibitory effect of IL-1 may be counterbalanced in the presence of RAR agonist since all RAR subtypes contain a retinoic acid response element (RARE) in their promoter region [26], which renders them highly inducible by these agonists. In our experimental conditions, we observed that ATRA induced a threefold increase in *RAR *mRNA levels in IL-1-stimulated cells (data not shown), therefore suggesting that our biological system was suitable to study the pharmacological properties of agonists of RAR or RXR subtypes.

In IL-1-stimulated synovial fibroblasts, we demonstrated that ATRA reduced the IL-1-induced expression and release of IL-6 whereas the RXR agonist, BMS-649, was ineffective. For the RAR agonist, this result was consistent with its ability to reduce cytokine production by fibroblastic cells [27] or macrophages [28] challenged with various inflammatory stimuli. In the case of RXR agonist, the lack of efficacy of BMS-649 was not unexpected even if RXR agonists are well known to act as co-stimulators and were shown to potentiate the effect of PPAR (peroxisome proliferator-activated receptor) [29] or RAR [30] agonists rather than to display intrinsic anti-inflammatory properties. However, we also failed to demonstrate any potentiation of the suppressive effect of ATRA on IL-6 when it was used in combination with BMS-649, suggesting that activation of RXR played a minor role. As a consequence, ATRA could reduce IL-6 expression by activating RAR/RAR homodimers, which were shown to exist albeit less abundantly than RAR/RXR heterodimers [31], or by RAR-independent mechanisms. Complementary experiments with selective agonists of each RAR subtype (BMS-753 for RAR-α, BMS-453 for RAR-β, and BMS-961 for RAR-γ), or with RAR designed against siRNA, demonstrated that the suppressive effect of ATRA on IL-6 was RAR-independent in our cell type.

To search for signalling events able to drive the suppressive effect of ATRA on IL-6, we investigated the possible contribution of upstream MAPKs thought to be responsive to IL-1. We demonstrated that, as expected from the fibroblastic phenotype of our cell type, p38 MAPK [32] and JNK [33] were activated in response to IL-1 challenge. However, we further showed that activation of neither of these pathways was inhibited by ATRA. As a reduced phosphorylation of p38 was thought to contribute to the inhibitory effect of ATRA on IL-1-induced expression of cytokines in chondrocytes [34], these data highlight that ATRA may act in a cell-dependent manner. In contrast, we showed that phosphorylation of ERK_1/2 _was an early IL-1-mediated event in rat synovial fibroblasts and demonstrated that it was reduced significantly by ATRA. Activation of ERK_1/2 _was also reported in mouse [35] and RA [36] synovial fibroblasts challenged with IL-1 and this was shown to contribute to the production of IL-6 in response to inflammatory stimuli. In our experimental system, the MEK1 inhibitor PD-98059 reproduced the suppressive effect of ATRA on IL-6 at a concentration actively preventing the phosphorylation of ERK_1/2_. Taken together, these data strongly suggested that the inhibition of the ERK_1/2 _pathway by ATRA supported its suppressive effect on IL-1-induced production of IL-6.

The transcription factors NF-IL-6, AP-1, and NF-κB possess binding sites in the promoter of *IL-6 *but their respective contributions to the stimulating effect of IL-1 on *IL-6 *expression seem to be variable in RA synovial fibroblasts [37,38]. In our system, we demonstrated that IL-1 activated NF-IL-6, AP-1, and NF-κB pathways although with a different kinetics. Kinetics may explain some discrepancy between the published data with human cells since we confirmed a lack of activation of NF-IL-6 or c-Jun after 30 minutes of stimulation with IL-1 [37] but a strong activation of both pathways after 4 hours of stimulation [38]. At the time of their optimal activation, we demonstrated that ATRA suppressed NF-IL-6 and AP-1 pathways but not NF-κB pathway and this effect was reproduced by the ERK_1/2 _inhibitor PD-98059. In contrast, the inhibitory effect of PD-98059 showed that AP-1 was activated by ERK_1/2 _in synovial fibroblasts [39,40] and could contribute to the enhanced production of IL-6 in response to IL-1 [38]. This result was highly consistent with the regulation of MMP-1 by IL-1 in rabbit synovial fibroblasts, where the ERK_1/2_, but not the p38, pathway accounted for the phosphorylation and activation of c-Jun [39]. The third transcription factor, NF-IL-6, can be activated by a lot of biological signals but contains a highly conserved phosphorylation site for MAPKs [41], which regulates its nuclear translocation in response to growth factors [42]. Our data demonstrated that activation of NF-IL-6 depended on activation of ERK_1/2_, as was reported for the differentiating effect of adiponectin on preadipocyte fibroblasts [43]. Such blockade of NF-IL-6 by PD-98059 inducing the inhibition of IL-6 levels was shown in Kaposi sarcoma cells [44]. Thus, we provide evidence that ERK_1/2 _contributed to the IL-1-induced activation of AP-1 and NF-IL-6 in synovial fibroblasts. In addition, our data suggest that the suppressive effect of ATRA on these transcription factors may be supported by inhibition of ERK_1/2 _phosphorylation. This mechanism is consistent with the ability of retinoids to mediate most of their anti-inflammatory effects by reducing activation of the AP-1 (for a review, see [45]) or NF-IL-6 [44] pathways, although it remains to be confirmed in other cell types.

## Conclusion

The present work demonstrated that ATRA, but several selective RAR agonists, displayed a powerful inhibitory potency on IL-1-induced expression and release of IL-6 by synovial fibroblasts. Despite the presence of all RAR and RXR subtypes, except RXR-γ, this suppressive effect was RAR-independent and not sensitive to RXR activation. Among MAPKs, ATRA reduced the phosphorylation of ERK_1/2 _and the activation of AP-1 and NF-IL-6 pathways, but not of NF-κB. These effects were reproduced by the MEK1 inhibitor PD-98059, suggesting that inhibition of the ERK_1/2 _pathway supported the suppressive potency of ATRA on transcription factors and IL-6 release. The present data underline that a decreased synthesis of IL-6 by synovial fibroblasts can contribute to the anti-arthritic potency of ATRA in animal models, as was suggested for the inhibitory effect of Am-80 in human osteoblast-like cells [46]. Nonetheless, this cytokine-suppressive potency was independent of RAR, indicating that alternative mechanisms, such as modulation of histone deacetylase activity [39], or retinoylation of kinases upstream of the ERK_1/2 _pathway [47,48] could support the anti-inflammatory effect of other retinoids.

## Abbreviations

AA: adjuvant arthritis; AP-1: activator protein-1; ATRA: all-*trans *retinoic acid; bp: base pair; CIA: collagen-induced arthritis; Ct: threshold cycle; DMSO: dimethyl sulfoxide; ELISA: enzyme-linked immunosorbent assay; ERK: extracellularly regulated kinase; IL: interleukin; JNK: c-Jun N-terminal kinase; MAPK: mitogen-activated protein kinase; MEK1: mitogen-activated protein extracellularly regulated kinase kinase 1; MMP: matrix metalloprotease; MTT: 3-(4,5-dimethylthiazol-2-yl)-2,5-diphenyl-2*H*-tetrazolium bromide; NF-IL-6: nuclear factor of interleukin-6; NF-κB: nuclear factor-kappa-B; PCR: polymerase chain reaction; RA: rheumatoid arthritis; RAR: retinoic acid receptor; RARE: retinoic acid response element; RE: responsive element; RP29: ribosomal protein S29; RXR: retinoid X receptor; SAPK: stress-activated protein kinase; siRNA: small interfering RNA; TNF-α: tumor necrosis factor-alpha.

## Competing interests

The authors declare that they have no competing interests.

## Authors' contributions

MKi performed all experiments and drafted the manuscript. MKo contributed to the study of RAR or RXR subtype expression and statistical analysis. SS performed the TransAm™ assays and contributed to the experiments with PD-98059. PN supervised the study design and the drafting of the manuscript. J-YJ contributed to the study design, data analysis, and final presentation of the manuscript. AB conceived the study, participated in its design and data analysis, and drafted the manuscript. All authors read and approved the final manuscript.
